# A hospital based registry of juvenile idiopathic arthritis in Norway

**DOI:** 10.1186/1546-0096-12-S1-P203

**Published:** 2014-09-17

**Authors:** Trude M Ingebrigtsen, Berit Flatø, Torhild Garen, Helga Sanner

**Affiliations:** 1 Norwegian Competence Centre of Paediatric and Adolescent Rheumatology, Department of Rheumatology, Oslo University Hospital, Oslo, Norway; 2 Department of Rheumatology, Oslo University Hospital, Oslo, Norway; 3 Faculty of Medicine, University of Oslo, Oslo, Norway

## Introduction

In 1999 a registry of patients with juvenile idiopathic arthritis (JIA) and juvenile onset connective tissue diseases was established at Rikshospitalet, Oslo University Hospital. The purpose of the registry is to initiate clinical, epidemiologic and laboratory research projects.

## Objectives

To evaluate clinical characteristics in patients with JIA registered in the hospital based registry.

## Methods

The registry is based on written informed consent. JIA patients were classified using ICD-10 codes and they were registered once. Gender, date of registration and year for disease onset was recorded, in addition to onset type, number of active joints and physician’s global assessment of disease activity (VAS 0-100 mm).

## Results

A total of 1069 JIA patients were registered. Mean age at inclusion was 9 years, and 65% were female. The distributions in various ICD-10 subgroups are shown in table 1. The most frequent subgroups were pauciarticular and polyarthritis (seronegative).

Physician’s global was higher in girls (n=664) than in boys (n=353) in the group as whole; median 16 (IQR 4-33) versus median 13 (IQR 3-28); p=0.049. When comparing physician’s global between JIA subgroups, polyarthritis had higher than both pauciarticular arthritis; p=0.001, and systemic arthritis; p=0.024 (table 1). In 992 patients data on onset type was available. Of these 66% had pauci, 26% poly, 6% systemic and 2% unknown onset type. In 854 patients data on active joint count at time of registration was available. In the group as whole 42% had none, 24% one, 13% two, 6% three, 3% four and 12% five or more active joints.

**Table 1:** Distribution of ICD-10 code, gender, age, disease duration and physician's global. (N=1069)

ICD-10 code	Diagnosis	n (%)	Female%	Mean (SD) age at inclusion	Mean (SD) age at disease onset	Median (IQR) disease duration (years)	Median (IQR) physician's global
M08.0	Juvenile rheumatoid arthritis	55 (5)	86	11 (4)	8 (5)	2 (1,5)	16 (3,27)

M08.1	Juvenile ankylosing sponydlitis	21 (2)	29	15 (2)	11 (2)	3 (1,6)	13 (5,31)

M08.2	Juvenile arthritis with systemic onset	77 (7)	53	8 (5)	5 (4)	2 (1,4)	12 (3,49)

M08.3	Juvenile polyarthritis (seronegative)	269 (25)	75	10 (5)	5 (5)	2 (1,6)	21 (7,43)

M08.4	Pauciarticular juvenile arthritis	545 (51)	64	8 (5)	5 (4)	2 (1,5)	12 (3,26)

M08.8	Other juvenile arthritis	26 (3)	54	13 (3)	10 (4)	2 (0,3)	27 (15,35)

M08.9	Juvenile arthritis, unspesified	53 (5)	53	12 (5)	9 (5)	2 (1,5)	14 (4,31)

M09.0	Juvenile arthritis in psoriasis	23 (2)	48	13 (3)	10 (4)	2 (1,4)	19 (5,30)

Total		1069 (100)	65	9 (5)	6 (5)	2 (1,5)	15 (4,31)

## Conclusion

The age at onset and gender distribution is as expected. Girls had higher physician’s global than boys and those with polyarthritis had higher physician’s global than both systemic and pauciarticular arthritis. Ideally the register should have been based on the ILAR criteria instead of the ICD-10 codes. We consider the three subgroups pauci, poly and systemic to be the most consistent with the ILAR criteria. The registry will be an important database for studies concerning outcome in JIA.

## Disclosure of interest

None declared.

